# The Utilization by *Bacteroides* spp. of a Purified Polysaccharide from Fuzhuan Brick Tea

**DOI:** 10.3390/foods13111666

**Published:** 2024-05-26

**Authors:** Jiameng Shi, Wangting Zhou, Guijie Chen, Wei Yi, Yi Sun, Xiaoxiong Zeng

**Affiliations:** College of Food Science and Technology, Nanjing Agricultural University, Nanjing 210095, China; 2021108059@stu.njau.edu.cn (J.S.); 2019208019@njau.edu.cn (W.Z.); guijiechen@njau.edu.cn (G.C.); 2022108024@stu.njau.edu.cn (W.Y.); sunyi01@njau.edu.cn (Y.S.)

**Keywords:** Fuzhuan brick tea, polysaccharide, *Bacteroides*, transcriptome

## Abstract

In the present study, four *Bacteroides* species that could degrade Fuzhuan brick tea polysaccharide-3 (FBTPS-3) were isolated from human feces and identified to be *Bacteroides ovatus, B. uniformis*, *B. fragilis and B. thetaiotaomicron*. The four *Bacteroides* species showed growth on FBTPS-3 as the carbon source, and *B. ovatus* showed the best capability for utilizing FBTPS-3 among the four species since *B. ovatus* could utilize more FBTPS-3 during 24 h fermentation. Moreover, the four *Bacteroides* species could metabolize FBTPS-3 and promote the production of acetic, propionic and isovaleric acids. Transcriptome analysis of *B. ovatus* revealed that 602 genes were up-regulated by FBTPS-3, including two carbohydrate-active enzyme clusters and four polysaccharide utilization loci (PULs). The PUL 1 contained GH28 family that could hydrolyze rhamnogalacturonan and other pectic substrates, which was in line with our previous work that rhamnose and galacturonic acid were the main component monosaccharides of FBTPS-3. Collectively, the results suggested that FBTPS-3 could be utilized by *Bacteroides* spp., and it might be developed as a promising prebiotic targeting Bacteroidetes in intestinal environment.

## 1. Introduction

Trillions of intestinal bacteria inhabit in the gut, and approximately 30% of them are from the Bacteroidetes phylum [1]. *Bacteroides*, the dominant genus member of Bacteroidetes, can produce a large number of carbohydrate-active enzymes (CAZymes), making itself thrive on various carbohydrate nutritional resources [2]. *Bacteroides* dedicates up to 20% of its genome to the degradation and utilization of complex polysaccharides [3]. The genes encoding CAZymes involved in the glycan-binding, transport and hydrolysis are co-localized in clusters, which are so-called polysaccharide utilization loci (PULs) [4,5]. The PULs vary among the genomes of different *Bacteroide* species and can be up-regulated in response to different dietary glycans. For example, *B. dorei* possesses the PULs that encode the apparatus required for β-mannan-like glycan catabolism [6], and *B. thetaiotaomicron* A4 contains PULs responsible for the breakdown of galactans [7]. *B. thetaiotaomicron* encodes a PUL for the breakdown of β-2,6-linked fructan levan, while the associated PUL in *B. uniformis* lacks the enzyme for levan breakdown [8]. *B. ovatus* and *B. thetaiotaomicron* possess homologous PULs orchestrating the degradation of pectin rhamnogalacturonan II (RG II). However, *B. thetaiotaomicron* contains several unique PULs that enable the degradation of host mucin *O*-glycans, a phenotype absent in *B. ovatus* [9]. Therefore, elucidating how *Bacteroides* deploy PULs for the breakdown of specific glycan will provide us information on how the glycan regulate gut microbiota. The preference of different *Bacteroides* for carbon source depends on the PULs they possess.

Fuzhuan brick tea (FBT), with a unique flavor and mainly sold in the minority areas of northwest China, is one of the most representative post-fermented teas (dark teas) [10]. FBT contains many bioactive substances, such as polyphenols, polysaccharides, amino acids and alkaloids [11,12]. Among them, polysaccharides (about 8% weight of dried FBT) are the main components. It was found that polysaccharides from FBT (Fuzhuan brick tea polysaccharide, FBTPS) had excellent antioxidant activity and showed significant ameliorative effect on high-fat-diet-induced metabolic disorders in mice [13]. Our previous study also observed that Fuzhuan brick tea polysaccharide-3 (FBTPS-3), a main purified fraction of crude FBTPS, exhibited good antioxidant activity. Moreover, one recent study showed that both FBTPS-3 and the crude FBTPS could alleviate dextran sulfate sodium-induced colitis in mice by regulating intestinal barrier homeostasis, and the effect of FBTPS-3 was superior to that of crude FBTPS [14]. Therefore, FBTPS-3 is considered as a natural active substance with development prospects.

Intestinal flora has vital effects for a host, such as promotion of energy metabolism, boost of immunologic maturation and regulation of nutrient absorption [15,16]. In our previous study, the results of in vitro-simulated digestion showed that the crude FBTPS were barely degraded in the upper gastrointestinal tract, but they could be fermented by intestinal microorganisms located in the colon while they could promote the production of short-chain fatty acids (SCFAs). It was speculated that the main way FBTPS-3 performed its biological activity was to be metabolized by gut microbes and promote the production of beneficial products [17]. Moreover, in vitro fermentation revealed that FBTPS-3 could modulate the gut community profile, and at genus level, FBTPS-3 significantly enriched *Bacteroides*, *Prevotella*, *Paraprevotella*, *Dialister* and *Sutterell*, among which *Bacteroides* was the important glycan degrader [18]. Therefore, the interaction between FBTPS-3 and *Bacteroides* might play an important role in host health. However, previous experiments were mainly focused on the regulatory effects of FBTPS-3 on mixed bacteria; the utilization of FBTPS-3 by specific *Bacteroides* species is still unclear. Therefore, in the present study, four *Bacteroides* species were isolated and identified by using *Bacteroides* bile esculin (BBE, a medium used for *B. fragilis* homologue screening) plates [19], and their utilization capability for FBTPS-3 was then investigated in order to prove that *Bacteroides* strains were the key degrader of FBTPS-3. Moreover, we employed transcriptome analysis to discover possible PULs of *B. ovatus* which might be involved in FBTPS-3 degradation. This study could provide theoretical guidance on the targets of FBTPS-3 bio-efficacy effects.

## 2. Materials and Methods

### 2.1. Materials and Chemical Reagents

FBT was obtained from the Baishaxi Tea Factory (Yiyang, Hunan, China), and the chemical reagents and media used for the experiments were purchased from Nanjing Shoude Biological Technology Co., Ltd. (Nanjing, China). DEAE Sepharose Fast Flow was bought from GE Healthcare Life Science (Pittsburgh, PA, USA).

### 2.2. Preparation of FBTPS-3

FBTPS-3 was prepared according to our reported method [18]. Briefly, FBT was firstly extracted with 95% (*v*/*v*) ethanol solution to remove the small molecules such as polyphenols. Then, the residues were extracted with pure water, and the supernatants were collected, concentrated, mixed with four volumes of anhydrous ethanol, and kept overnight. The resulting precipitates were collected, and the proteins in the precipitates were removed by Sevag method [20]. The precipitates were re-dissolved in pure water, followed by the process of dialysis and freeze-drying, affording crude FBTPS. The crude FBTPS was dissolved in pure water and loaded onto a DEAE Sepharose Fast Flow column (2.6 × 50 cm). The column was washed with a stepwise gradient of sodium chloride solutions (0, 0.1, 0.3 and 0.5 M). The fractions eluted with 0.3 M of sodium chloride solution were combined, dialyzed, concentrated, and named as FBTPS-3. In the present study, the physicochemical properties and monosaccharide composition of FBTPS-3 were determined to be consistent with those previously published [18].

### 2.3. Isolation of Bacteroides Species

In vitro fermentation of FBTPS-3 was firstly carried out according to the reported method [21]. In brief, the basal nutrient medium (2.0 g/L of yeast extract, 2.0 g/L of peptone, 0.1 g/L of NaCl, 0.01 g/L of MgSO_4_·7H_2_O, 0.04 g/L of KH_2_PO_4_, 0.04 g/L of K_2_HPO_4_, 0.01 g/L of CaCl_2_, 2 g/L of NaHCO_3_, 0.02 g/L of heme, 0.5 g/L of cysteine-HCl, 0.5 g/L of bile salt, 2.0 mL/L of Tween 80, 1.0 mL/L of 1% resazurin, 10 μL/L of vitamin K_1_) supplied with FBTPS-3 (2.0 g/L) was prepared. The fresh feces from three healthy volunteers who had not taken antibiotics and probiotics for the last three months were collected, and the fecal slurry (10%, *w*/*v*) was obtained by suspending feces in sterilized normal saline. Then, 1 mL of fecal slurry was added to 10 mL of basic medium containing FBTPS-3, and the mixture was inoculated in an anaerobic incubator (10% H_2_, 5% CO_2_ and 85% N_2_). After 24 h fermentation, the broth was diluted by sterilized normal saline with a final concentration of 10^−1^, 10^−2^, 10^−3^, 10^−4^ and 10^−5^, respectively. The resulting solution was applied to BBE plates with three replicates. After 48 h incubation, suspicious colonies were selected for scribing based on the appearance characteristics. After purification three times, the screened isolates were stained with Gram stain, and the Gram-negative isolates were preserved with 30% (*v*/*v*) glycerol and stored at −80 °C.

### 2.4. Identification of Bacteroides Species

The preserved bacterial solution samples were activated and transferred into Eppendorf vials individually, and then they were sent to Nanjing Paisano Technology Co., Ltd. (Nanjing, China) for identification based on 16S rDNA sequencing [19]. The DNA was exacted and hybridized. The PCR reaction conditions were set as the following. The forward and reverse primers were 27F (5′-AGAGTTTGATCCTGGCTCAG-3′)/1492R (5′-GGTTACCTTGTTACGACTT-3′). The PCR reaction system consisted of 10 μL of 2 × Taq PCR Master Mix, 1 μL of forward primer (10 μM), 1 μL of reverse primer (10 μM), 1 μL of template DNA, and 7 μL of ddH_2_O. The PCR amplification parameters were set as 95 °C pre-denaturation for 5 min, 95 °C denaturation for 30 s, annealing at 54 °C for 30 s, extension at 72 °C for 1 min, and cycling 35 times, then terminal extension at 72 °C for 5 min. The PCR products were sequenced, and the sequencing results were spliced to remove the unreliable sequences at both ends. The sequence comparison was performed using Nucleotide BLAST (http://blast.ncbi.nlm.nih.gov/Blast.cgi, accessed on 10 April 2023). The phylogenetic relationship and phylogenetic status of the screened strains and the known strains were compared, and if the homology was more than 99%, it could be recognized as the same type of bacteria. The phylogenetic tree was established by Mega X software 11. In this study, four *Bacteroides* species including *B. ovatus* (BO), *B. uniformis* (BU), *B. fragilis* (BF), and *B. thetaiotaomicron* (BT) were isolated and identified. The GenBank ID of four *Bacteroides* species are listed in Table 1.

### 2.5. Determination of Growth Curve of Bacteroides Species

Four *Bacteroides* strains were cultivated in Gifu anaerobic medium (GAM). Each species harvested from the exponential growth phase was re-suspended in sterilized normal saline until optical density at 600 nm (OD_600_) reached 0.6. Then, 0.2 mL of bacterial suspension was added to 10 mL of *Bacteroides* minimal medium (BMM, 10 g/L of tryptone, 17.5 g/L of bovine heart infusion powder, 5 g/L of NaCl, 2.5 g/L of Na_2_HPO_4_·12H_2_O, 0.5 g/L of cysteine-HCl, 1 g/L of NaHCO_3_, 20 mg/L of heme) supplied with FBTPS-3 (2 g/L or 5 g/L) [22]. During the cultivation, 0.3 mL of sample was taken every 4 h up to 36 h, and the growth was monitored by measuring OD_600_ with a micro-plate reader (BioTek Instruments Inc., VT, USA).

### 2.6. Analysis of FBTPS-3 Utilization by Bacteroides Members

To determine the utilization of FBTPS-3 by *Bacteroides* species, 0.2 mL of culture of the strain was adjusted to OD_600_ 0.6 in sterilized normal saline and then added to 10 mL of BMM containing 2 g/L of FBTPS-3. Three replicates were made for each strain, and the fermentation was performed in an anaerobic incubator. During the fermentation period, samples were taken at 0, 4, 8, 12, and 24 h, respectively, and the supernatant was collected by centrifugation at 8000 rpm for 5 min. The relative carbohydrates content for the fermentation supernatant was determined using the phenol–sulfuric acid method. The content of reducing sugars was determined by 3,5-dinitrosalicylic acid (DNS) colorimetric method [23]. The molecular weight (Mw) distribution was determined using high performance gel permeation chromatography (HPGPC) on an Agilent 1200 series instrument equipped with a TSK G4000PW_XL_ column (7.8 × 300 mm) and refractive index detector. The mobile phase was 0.1 M o Na_2_SO_4_ and 0.01 M of phosphate buffer (pH 6.8). The column temperature was set as 35 °C, and the injection volume was 20 μL. Before HPGPC analysis, the fermentation supernatant was mixed with four volumes of absolute ethanol. Subsequently, the sediment was collected, re-dissolved in pure water, and passed through a 0.45 μm filter.

### 2.7. Determinations of Contents of SCFAs

The contents of SCFAs for the fermentation supernatant were measured by gas chromatography (GC). A series of mixed standard solutions of SCFAs (acetic, propionic, n-butyric, isobutyric, n-valeric, and isovaleric acids) were prepared, and 2-ethylbutyric acid was used as the internal standard. The samples were mixed with equal volume of the internal standard, and the mixture was passed through an aqueous 0.45 μm filter membrane and analyzed on an Agilent 6890 N GC system equipped with a flame ionization detector (FID) and an HPINNOWAX column (30 m × 0.25 mm × 0.25 μm, Agilent), as reported previously [24]. In brief, nitrogen was set as the carrier gas at a follow rate of 19 mL/min, and the flow rates of air, nitrogen, and hydrogen were 260, 30, and 30 mL/min, respectively. The initial column temperature was set as 100 °C for 1 min, then gradually increased to 180 °C at a speed of 5 °C/min, and finally further maintained at 180 °C for 4 min. The injection volume was 1 μL.

### 2.8. Transcriptome Analysis of BO

BO was grown in triplicate on either glucose or FBTPS-3 as a sole carbon source, and the cells were harvested during the mid-log phase. The medium containing glucose (Glc) was set as the control group (GLC group), and the medium containing FBTPS-3 was set as the experimental group (FBTPS-3 group). Total RNA was extracted by an RNA isolation kit (Vazyme Biotech Co., Ltd., Nanjing, China). After quality assessment, an RNA-seq library was built according to the previously described method [25]. Samples were sequenced at Nanjing Paisano Technology Co., Ltd. by using the Illumina HiSeq 2500 platform. Reads from the samples were mapped onto the *B. ovatus* ATCC 8483 reference genomes (NCBI RefSeq accession number: NZ_CP012938.1) using a highly stringent cutoff. DESeq (version 1.18.0) was used to screen for differentially expressed genes (DEGs). Genes were considered significantly differentially expressed when the absolute log2 fold change (FC) of the transcription in the FBTPS-3 group relative to that in the GLC group was beyond 1.0 and the false-discovery rate value was below 0.05. CAZymes annotation of the differential genes was carried out using dbCAN-PUL (https://bcb.unl.edu/dbCAN2/blast.php, accessed on 27 March 2024).

### 2.9. Statistical Analysis

The data are expressed as the mean ± standard deviation (SD). The data analysis was performed using SPSS 25.0 software (IBM). One-way analysis of variance (ANOVA) followed by Dunnett’s multiple comparison test was used to analyze whether there was a significant difference among different groups. Significant differences existed between the two groups when the *p* value was less than 0.05.

## 3. Results

### 3.1. Identification of Bacteroides Strains Isolated from the Human Feces

**Figure 1:**
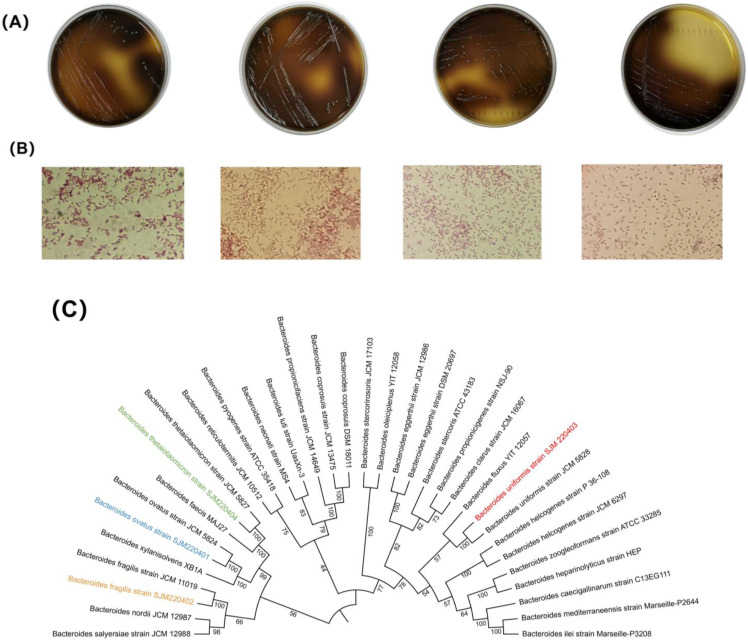
(**A**) The picture of *B. ovatus* (BO), *B. uniformis* (BU), *B. fragilis* (BF) and *B. thetaiotaomicron* (BT) grew on BBE plates, respectively. (**B**) The Gram-staining microscope pictures of BO, BU, BF and BT, respectively. (**C**) Phylogenomic tree of the genus Bacteroidetes and four isolated strains.

To explore the regulating mechanism of FBTPS-3 on *Bacteroides* strains, we isolated and identified four *Bacteroides* strains from human feces including BO (SJM 220401), BU (SJM 220403), BF (SJM 220402) and BT (SJM 220404). As shown in Figure 1, the isolated *Bacteroides* species were grayish white with smooth edges on BBE plates (Figure 1A) and Gram-negative bacteria with a short rodlike shape (Figure 1B). The phylogenetic tree is shown in Figure 1C. It could be found that SJM 220401, SJM 220403, SJM 220402 and SJM 220404 shared high similarity with *B. ovatus* JCM 5824, *B. uniformis* JCM 5828, *B. fragilis* JCM 11019 and *B. thetaiotaomicron* JCM 5827, respectively.

### 3.2. Utilization Capability of Four Bacteroides Species on FBTPS-3 in Single Culture

**Figure 2:**
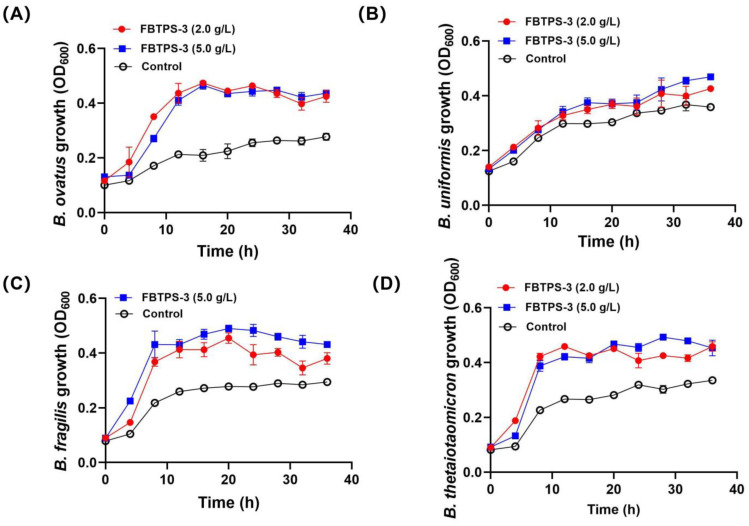
The growth curves of four *Bacteroides* strains growing on 2.0 g/L or 5.0 g/L Fuzhuan brick tea polysaccharide-3 (FBTPS-3). The growth curve of BO (**A**), BU (**B**), BF (**C**) and BT (**D**).

As shown in Figure 2A–D, BO, BU, BF and BT showed growth on FBTPS-3 at both concentrations of 2.0 g/L and 5.0 g/L. Notably, the maximum OD_600_ values of BO, BF and BT were higher than that of BU, indicating that the growth-promoting effect of FBTPS-3 for BU was weaker than that for the other three strains. Moreover, the results also showed that the growth of the four strains was not dependent on the amount of FBTPS-3 provided. Thus, the subsequent experiments were carried out with FBTPS-3 at a concentration of 2.0 g/L.

**Figure 3:**
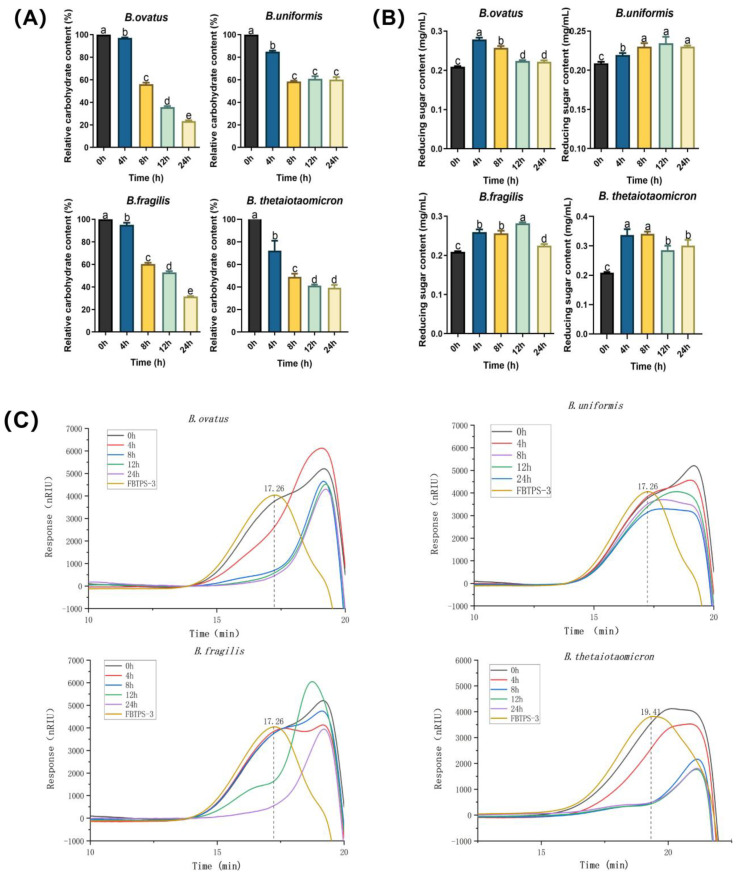
Utilization of FBTPS-3 by four *Bacteroides* strains. (**A**) The change in carbohydrate content. (**B**) The change in reducing sugar content. (**C**) Molecular weight (Mw) change determined by high performance gel permeation chromatography (HPEGC). Different letters (a, b, c, d) indicate significant differences (*p* < 0.05) for the carbohydrate content or the reducing sugar content among different time points.

To verify the utilization capability of four *Bacteroides* species on FBTPS-3, the contents of total carbohydrates and reducing sugars in culture supernatants were determined. As shown in Figure 3A, the contents of total carbohydrates in culture supernatants of BO, BU, BF and BT reduced in a time-dependent manner, further suggesting that all four *Bacteroides* species could grow on FBTPS-3. Additionally, after 24 h culture, 76.6 ± 0.6%, 39.8 ± 2.1%, 68.5 ± 0.3% and 60.6 ± 2.5% of FBTPS-3 were consumed by BO, BU, BF and BT, respectively. The results indicated that BO had the best capability for utilizing FBTPS-3 among the four species as BO could utilize more FBTPS-3 after 24 h fermentation compared with the other three *Bacteroides* species. Moreover, as displayed in Figure 3B, the reducing sugars contents in culture supernatants of BO, BF and BT were increased after 4 h fermentation and then decreased after 8 h fermentation. It was speculated that these changes might be correlated to the FBTPS-3-degrading strategy of those *Bacteroides* species during fermentation. FBTPS-3 was firstly degraded into fragments outside the cell, which exposed some reducing ends, and the fragments were subsequently transported to the periplasm and further degraded and utilized by bacteria [26,27,28]. In addition, the reducing sugars content in culture supernatants of BU was increased after 4 h fermentation but then kept stabilized after 8 h fermentation, further indicating that BU had poor utilization capability for FBTPS-3 compared with the other three *Bacteroides* species.

The changes in HPGPC profiles of culture supernatants are shown in Figure 3C. The retention time of FBTPS-3 in the fermentation supernatants of BO, BU and BF was 17.26 min, while the retention time of FBTPS-3 in the BT fermentation supernatant was 19.41 min. The area of the peak of FBTPS-3 gradually decreased in a time-dependent manner after the fermentation by the four *Bacteroides* strains, and the retention time of the peak of FBTPS-3 also shifted back gradually [29]. Moreover, it could be seen that the relative peak of FBTPS-3 changed more rapidly when FBTPS-3 was utilized by BO or BT compared with BU and BF. The results indicated that the four *Bacteroides* strains were able to metabolize FBTPS-3 into small fragments. Notably, after 24 h fermentation, the area of the peak of FBTPS-3 in culture supernatants of BU was larger than that of BO, BF or BT, further suggesting that the FBTPS-3-degrading capability varied in those four *Bacteroides* species.

### 3.3. Production of SCFAs by four Bacteroides Species Grew on FBTPS-3

**Figure 4:**
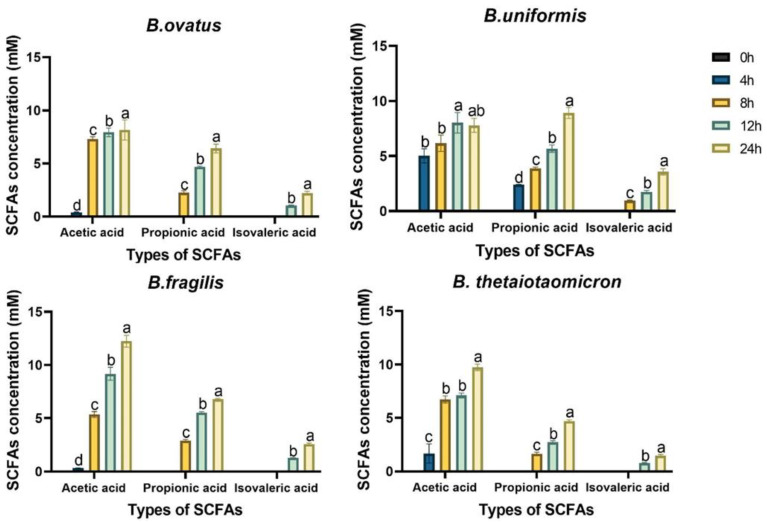
Concentrations of short-chain fatty acids (SCFAs) during four *Bacteroides* strains growing on FBTPS-3. Different letters (a, b, c, d) indicate significant differences (*p* < 0.05) for each SCFA among different time points.

As shown in Figure 4, the concentrations of SCFAs in culture of BO, BU, BF and BT in medium containing FBTPS-3 were determined. Four *Bacteroides* species could metabolize FBTPS-3 and promote the production of acetic, propionic and isovaleric acids. During the fermentation, acetic acid was the predominant SCFA, followed by propionic and isovaleric acids. After 24 h fermentation, the concentrations of acetic acid in the culture supernatants of BO, BU, BF and BT were 8.15 ± 0.95, 7.78 ± 0.64, 12.23 ± 0.55 and 9.72 ± 0.30 mM, respectively. The concentrations of propionic acid in the culture supernatants of BO, BU, BF and BT were 6.40 ± 0.42, 8.90 ± 0.49, 6.76 ± 0.09 and 4.68 ± 0.12 mM, respectively, and the concentrations of isovaleric acid reached 2.20 ± 0.18, 3.56 ± 0.28, 2.58 ± 0.09 and 1.46 ± 0.12 mM, respectively, for BO, BU, BF and BT.

### 3.4. Up-Regulated PULs and CAZyme Clusters in the Transcriptome of BO

According to the results of the above experiments, BO showed the best capability for FBTPS-3 utilization. Therefore, BO was selected for transcriptome analysis, aiming to obtain the key PULs involved in the process of FBTPS-3 degradation. After the fermentation of Glc and FBTPS-3, respectively, the gene expression profiles of the two groups were compared to find the DEGs.

**Figure 5:**
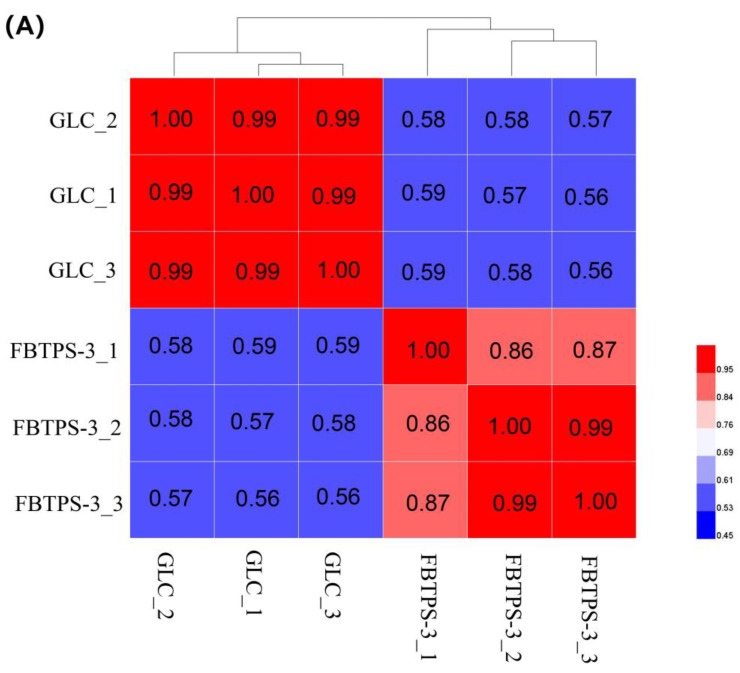

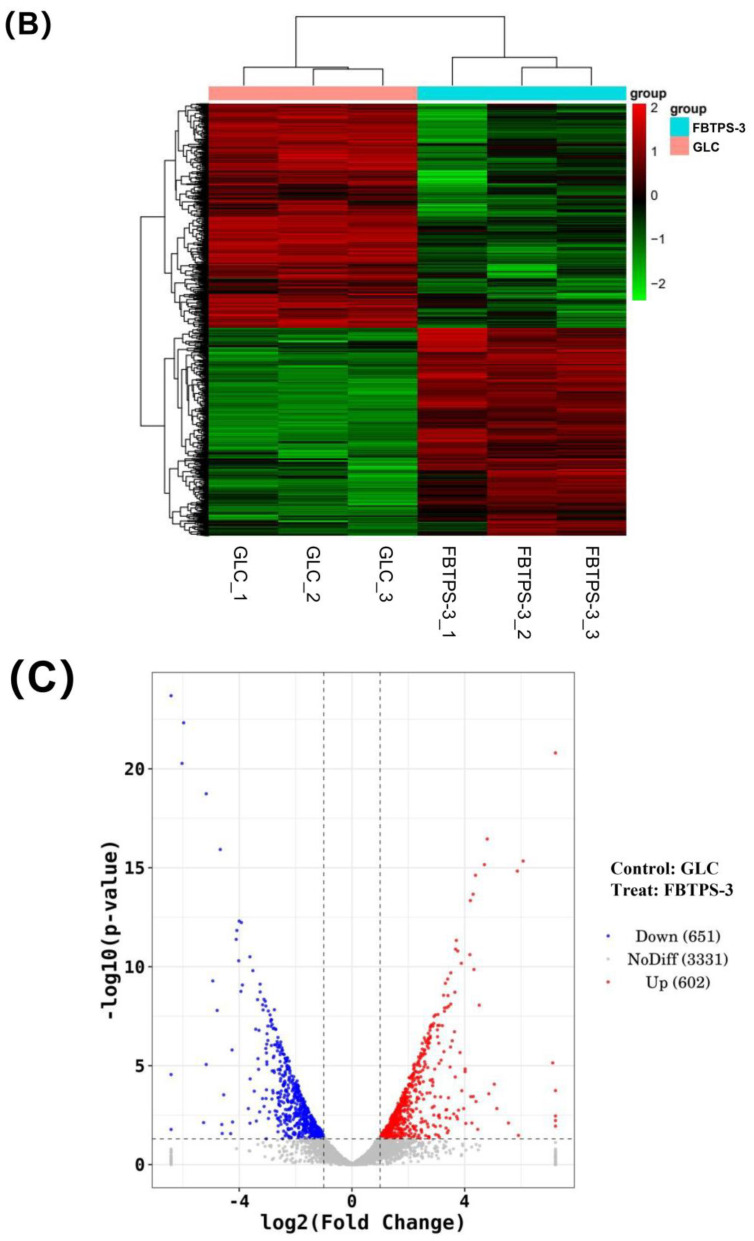
(**A**) Sample correlation analysis of the FBTPS−3 and GLC groups. (**B**) Differential clustering analysis of the FBTPS−3 and GLC groups. (**C**) Volcano plot of differentially expressed genes between the FBTPS−3 and GLC groups.

In the correlation analysis, a Pearson coefficient value between 0.8 and 1 indicates a good correlation between replicates. As exhibited in Figure 5A, the Pearson coefficient value for the replicates of FBTPS-3 group or GLC group was between 0.8 and 1, indicating that there was good reproducibility of the experiment. Next, we applied a cutoff of Log2FC ≥ 1 and FDR < 0.05 in expression in the FBTPS-3 group compared with the GLC group. Heatmap analysis showed obvious distinctions in gene expression between the two groups (Figure 5B). Moreover, as displayed in Figure 5C, 602 genes were up-regulated in the FBTPS-3 group compared with the GLC group. In contrast, 651 genes were down-regulated in the FBTPS-3 group compared with the GLC group.

**Figure 6:**
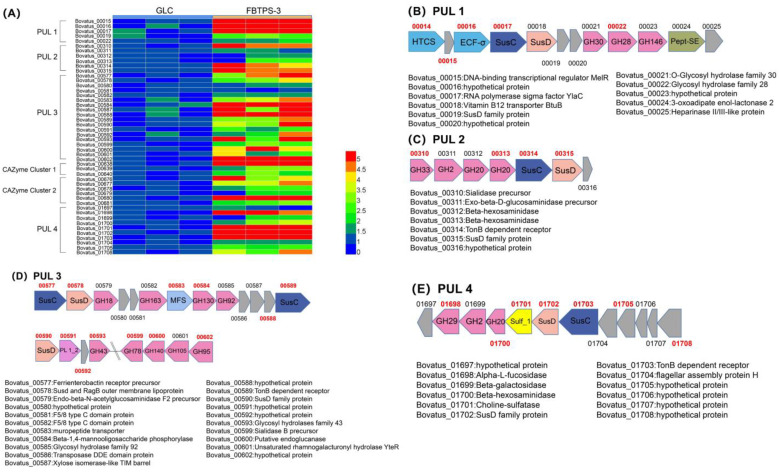
(**A**) Heatmap of up-regulated genes in the up-regulated polysaccharide utilization loci (PULs) and carbohydrate-active enzyme (CAZyme) clusters. (**B**) Schematic of PUL 1. (**C**) Schematic of PUL 2. (**D**) Schematic of PUL 3. (**E**) Schematic of PUL 4.

**Figure 7:**
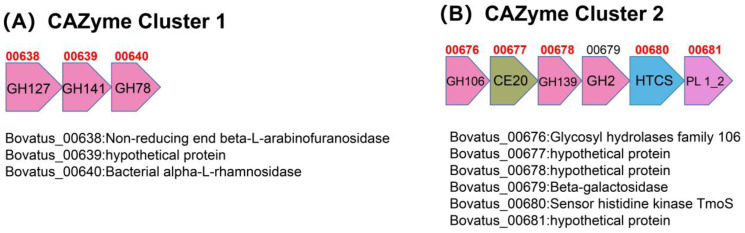
The schematic of CAZyme cluster 1 (**A**) and cluster 2 (**B**).

Notably, as it is shown in Figure 6A, the up-regulated genes were annotated against the dbCAN2 database and were successfully arranged into four up-regulated PULs (Figure 6B–E) as well as two up-regulated CAZyme clusters (Figure 7). There were also many up-regulated glycoside hydrolase (GH) family, carbohydrate esterase (CE) family, etc. These up-regulated CAZyme clusters and enzymes were not among the proven PULs, but they could also indicate to some extent that there were many hydrolytic enzymes with elevated expression when FBTPS-3 was utilized by BO.

As shown in Figure 6B–E, PUL1 (Bovatus_00014-00025) contained 12 genes, with hydrolase genes GH30, GH28 and GH146, in which the expression of GH28 was significantly up-regulated, and 00022 protein is a polygalacturonase that coincidentally corresponds to the polysaccharide structure [30]. PUL2 (Bovatus_00310-00316) contained seven genes, among which there were three hydrolases (GH33, GH2 and GH20). PUL3 (Bovatus_00577-00602) contained 26 genes, among which there were two pairs of Sus C and Sus D, and contained several hydrolases, a larger PUL. PUL4 (Bovatus_01697-01708) contained 12 genes with three hydrolytic enzyme classes (GH29, GH2 and GH20).

## 4. Discussion

FBTPS, one kind of the most important bioactive macromolecules in FBT, could regulate the composition of gut microbiota and promote the growth of *Prevotella* and *Bacteroides* [17]. As our previous report, four polysaccharide fractions including FBTPS-1, FBTPS-2, FBTPS-3 and FBTPS-4 were isolated from crude FBTPS by using anion-exchange chromatography. Among them, FBTPS-2 mainly increased the relative abundance of *Prevotella* in gut microbiota, whereas FBTPS-3 could increase not only the relative abundance of *Prevotella* but also that of *Bacteroides*. Our recent study showed that FBTPS-2 was a neutral polysaccharide with the backbone mainly formed by →4)-β-D-Gal*p*-(1→, →3)-β-D-Gal*p*-(1→ and →3)-β-D-Gal*p*-(1→4)-β-D-Galp-(1→ with branches on the *O*-6 of partial →3)-β-D-Gal*p*-(1→ mainly formed by →5)-α-L-Ara*f*-(1→ or →6)-β-D-Gal*p*-(1→, while FBTPS-3 was mainly composed of galacturonic acid (GalA), rhamnose (Rha), galactose (Gal), arabinose (Ara) and mannose (Man) in the molar ratio of 42.2:15.5:19.7:13.9:8.7, assumed to possess HG and RG Ⅰ domains [18,31]. The monosaccharide composition and structural information of FBTPS-3 were quite different from that of FBTPS-2. Therefore, we supposed that the modulating effects of those purified fractions of FBTPS on specific intestinal bacteria were closely related to their structural features. Similar with our results, the growth of *Bacteroides* was promoted by the polysaccharides that contained 1,4-linked D-Gal*p*A, such as HG and RG I. Moreover, some studies showed that the polysaccharides containing →4)-β-D-Gal*p*-(1→ could be a viable carbon source for the *Prevotella*, which was in line with results that FBTPS-2 primarily promoted the growth of *Prevotella* [32,33]. Furthermore, more studies such as methylation analysis and nuclear magnetic resonance (NMR) spectroscopy should be carried out to define the accurate structural information of FBTPS-3.

It has been reported that members of the genus *Bacteroides* are interested in the hydrolysis and utilization of complex glycans [3]. BO, BU, BF and BT are the common *Bacteroidetes* species inhabiting the intestinal tract and evolved to degrade a unique class of glycans. Genomic analysis exhibited that all the four species had the capacity to liberate and metabolize polymers. In this study, we found that the four species could degrade FBTPS-3 during fermentation in vitro, which was reflected by the changes in the relative carbohydrates content, reducing sugars content and HPGPC profiles (Figure 3A–C). Notably, BO, BF and BT could utilize 76.6%, 68.5% and 60.6% of FBTPS-3 in single culture after 24 h fermentation, respectively. In contrast, BU could consume less than 50% after fermentation. These results indicated that the ability to utilize FBTPS-3 varied considerably among BO, BU, BF and BT. Similar to our study, *B. uniformis* JCM 5828 could utilize laminarin, but it could not grow on agarose and porphyrin. That might be related to the PULs encoded in the genome of different *Bacteroides* strains, and they preferred different carbon sources [34]. Moreover, Centanni et al. reported that *B. ovatus*, *B. pectinophilus*, and *B. stercoris* showed a preference for the New Zealand spinach (*Tetragonia tetragonioides*) extract containing abundant homogalacturonan-type pectic polysaccharides, whereas *B. cellulosilyticus* and *B. intestinalis* showed a preference for the karaka berry (*Corynocarpus laevigatus*) extract containing abundant RG I pectic polysaccharides [35]. Additionally, it has been reported that BT could grow on three types of pectin (HG, RG I and RG II) [9]. We supposed that it might be related to the PULs encoded in the genome of different *Bacteroides* strains, and they preferred different carbon sources.

SCFAs are the main metabolites of the intestinal flora. SCFAs can not only provide energy, reduce the osmotic pressure of the intestinal environment, but also protect colonic epithelial cells, which play an important role in maintaining normal physiological functions of the intestine and protecting host health [36]. In this study, it could be found in Figure 4 that all the four *Bacteroides* species produced acetic, propionic and isovaleric acids during the degradation of FBTPS-3. In line with our previous in vitro fermentation study, it was found that FBTPS-3 could significantly promote the production of acetic and propionic acids, which might be related to the metabolism of FBTPS-3 by *Bacteroides* [18]. It has been shown that BO, BU, BF and BT could utilize carbon sources to produce acetic and propionic acids [37,38]. Acetic acid is the main product of the decomposition of pectin, and the fermentation of arabinogalactan produces large amounts of acetic and propionic acids, which is also in line with the monosaccharide composition of FBTPS-3 [39]. In addition, there was a small amount of isovaleric acid, which was the product of branched-chain amino acids, and it might be produced from the proteins in the medium [40]. As for *Bacteroides* spp., acetate is produced from pyruvate via acetyl-CoA, propionate is produced by succinate pathway. The results showed that acetic acid was produced first and in the highest yield, followed by propionic acid, and isovaleric acid was produced last and in the lowest yield, which was in line with the metabolic pathways of SCFAs [36,41]. And *B. dorei* had the same SCFA production profile when it grew on galactomannan or glucomannan [6].

As the results showed that BO had the best ability of utilizing FBTPS-3, we therefore selected BO for transcriptome analysis. Many CAZymes in *Bacteroides* are encoded in PULs. The transcriptome results showed that a large number of CAZymes, including four PULs and two CAZyme clusters, were up-regulated when BO grew on FBTPS-3 (Figure 6A). The high expression of Sus C and Sus D would occur in up-regulated PULs, as it showed in the results that almost all Sus C and Sus D in the four PULs have been up-regulated [22]. In PUL 1, there was a significant up-regulation of GH28, a glycoside hydrolase that could hydrolyze rhamnogalacturonan, which is in line with the monosaccharide composition of FBTPS-3. It has been reported that the degradation of HG was related to BT4108-BT4124, among which BT4123 was from the GH28 family. To some extent, it suggested that FBTPS-3 might have HG domains [9]. In PUL 2, the GH2 hydrolase is associated with pectin degradation [35]. There are many enzymes associated with pectin degradation in PUL 3, such as PL 1_2, GH43, GH28, CE19 and GH105, among which PL 1_2 and GH43 were significantly up-regulated. The PL 1 family can encode pectin cleavage enzymes, and the GH43 family can encode α-L-arabinosidase [42,43]. PLs could degrade HG into oligosaccharides in order to be transported to the periplasm. In line with this study, the degradation of arabinan was related to a specific PUL (BT0348-BT0369), in which BT0360 was GH43 hydrolase [44]. FBTPS-3 contained Ara, and Ara might be the side chain of FBTPS-3. To some extent, the transcriptome results suggested that FBTPS-3 might be a pectin polysaccharide. HG and RG Ⅰ domains were the most common pectin polysaccharide composition regions. The HG domain accounted for about 60% of the total pectin with the backbone of α-1,4-linked GalA residues [45,46,47]. The RG Ⅰ domain was composed of →4)-α-D-Gal*p*A-(1→2)-α-L-Rha*p*-(1→ repeats, and rhamnosyl residues could be substituted at the *O*-4 with some neutral sugar side chains [48,49]. Based on the results of the monosaccharide composition and transcriptome analysis, the backbone of FBTPS-3 might be →4)-α-D-Gal*p*A-(1→ and →4)-α-D-Gal*p*A-(1→2)-α-L-Rha*p*-(1→, and the branched chain might be composed of Gal, Ara and Glc connected at *O*-4 of →2)-α-L-Rha*p*-(1→. The precise structure of FBTPS-3 should be further confirmed by methylation analysis and NMR analysis to be able to cross-check with the transcriptome results.

## 5. Conclusions

In conclusion, in the present study, four *Bacteroides* species were isolated from human feces and identified to be BO, BU, BF and BT, and then their utilization for FBTPS-3 were investigated. Furthermore, it was found that the ability to utilize FBTPS-3 varied considerably among BO, BU, BF and BT, while BO was the best degrader. All the four *Bacteroides* species could promote the production of SCFAs (acetic, propionic and isovaleric acids) during the fermentation of FBTPS-3. Transcriptome analysis showed that the intervention of FBTPS-3 resulted in 602 genes up-regulated and 651 genes down-regulated in BO, involving four up-regulated PULs and two carbohydrase Clusters which provided a theoretical basis for the targeted regulation of *Bacteroides* by FBTPS-3. By analyzing the transcriptome in combination with monosaccharide composition, it could suggest that FBTPS-3 might contain HG and RG Ⅰ domains. Moreover, the key enzymes for FBTPS-3 degradation in BO still need to be discovered, and the key enzymes for FBTPS-3 degradation will be prepared by heterologous expression technology in the future. Meanwhile, there are fewer studies related to the degradation of complex glycans by BF, and the potential mechanisms of how to utilize FBTPS-3 by BF are still unclear. It can be further elucidated by transcriptomic techniques and further uncover the key PULs in BF for degradation of FBTPS-3.

## Figures and Tables

**Table 1:** The GenBank ID for the four *Bacteroides* species used in this study.

*Bacteroides* Species	GenBank ID
*B. ovatus*	OQ781165.1
*B. uniformis*	OR037385.1
*B. fragilis*	OQ781170.1
*B. thetaiotaomicron*	OR186207.1

## Data Availability

The original contributions presented in the study are included in the article, further inquiries can be directed to the corresponding author.
